# Current State of Understanding of the Role of PACAP in the Hypothalamo-Hypophyseal Gonadotropin Functions of Mammals

**DOI:** 10.3389/fendo.2020.00088

**Published:** 2020-03-06

**Authors:** Katalin Köves, Enikő Szabó, Orsolya Kántor, Andrea Heinzlmann, Flóra Szabó, Ágnes Csáki

**Affiliations:** ^1^Department of Anatomy, Histology and Embryology, Faculty of Medicine, Semmelweis University, Budapest, Hungary; ^2^Department of Conservative Dentistry, Faculty of Dentistry, Semmelweis University, Budapest, Hungary; ^3^Department of Molecular Embryology, Medical Faculty, Institute of Anatomy and Cell Biology, University of Freiburg, Freiburg, Germany; ^4^Department of Anatomy and Histology, University of Veterinary Sciences, Budapest, Hungary; ^5^Department of Pediatrics, Virginia Commonwealth University, Richmond, VA, United States

**Keywords:** PACAP, reproduction, mammals, male, female

## Abstract

PACAP was discovered 30 years ago in Dr. Akira Arimura's laboratory. In the past three decades since then, it has become evident that this peptide plays numerous crucial roles in mammalian organisms. The most important functions of PACAP are the following: 1. neurotransmitter, 2. neuromodulator, 3. hypophysiotropic hormone, 4. neuroprotector. This paper reviews the accumulated data regarding the distribution of PACAP and its receptors in the mammalian hypothalamus and pituitary gland, the role of PACAP in the gonadotropin hormone secretion of females and males. The review also summarizes the interaction between PACAP, GnRH, and sex steroids as well as hypothalamic peptides including kisspeptin. The possible role of PACAP in reproductive functions through the biological clock is also discussed. Finally, the significance of PACAP in the hypothalamo-hypophysial system is considered and the facts missing, that would help better understand the function of PACAP in this system, are also highlighted.

## Introduction

Pituitary adenylate cyclase activating polypeptide (PACAP) was discovered 30 years ago in Dr. Akira Arimura's laboratory in an effort to find the still undefined follicle stimulating hormone-releasing factor (FRF). It was discovered that a fraction of ovine hypothalamus was able to stimulate adenylate cyclase (AC) in rat primary anterior pituitary cell cultures (1). The peptide isolated from this tissue was then characterized. It is composed of 38 amino acids. The N-terminal sequence shows 68% homology with vasoactive intestinal polypeptide (VIP); however, its AC stimulating activity is at least 1,000 times greater than that of VIP. It was named PACAP38. PACAP belongs to the VIP/glucagon/secretin family. Its effect was tested on superfused pituitary cells and it demonstrated the ability to enhance the release of growth hormone (GH), prolactin (PRL), corticotropin (ACTH) and luteinizing hormone (LH). From fractions of hypothalamic tissues, a shorter form composed of 27 amino acids was also isolated, and it was named PACAP27. PACAP27 showed similar AC stimulating activity as PACAP38 (2). Further studies showed that PACAP stimulated AC in a multitude of other tissues. Its official name is now adenylate cyclase activating polypeptide (ADCYAP). It was also demonstrated that PACAP could stimulate other intracellular signal transduction mechanisms such as the phospholipase-C/protein kinase C (PLC/PKC) cascade (3). The human PACAP gene is located on chromosome 18p11.32 and encodes a 176–amino acid preproprotein, which comprises a 24–amino acid signal peptide (4). The cDNA encoding precursor of PACAP38 was successfully cloned from an ovine hypothalamic cDNA library by Kimura et al. (5).

The N-terminal domain of PACAP is responsible for the biological activity. Removal of the first amino acid (His^1^) of this domain reduced the potency and affinity of the peptide to its receptors. The removal of further amino acids decreased its affinity to the receptors to even greater degree. Finally, removal of the first five amino acids (PACAP6-38) resulted in an antagonist of PACAP (6).

PACAP is the most ancient and one of the most conserved member of the secretin superfamily (7). This peptide is found from invertebrates to humans. PACAP was demonstrated in various non-mammalian vertebrates such as different species of fish (8–11), frogs (12–17) and birds (18, 19). PACAP was able to stimulate AC in frog anterior pituitary cells (13, 14, 20).

Several important papers have reviewed the distribution of PACAP and its receptors, and the most important roles including the significance of PACAP in the reproductive functions (21–24). In the present paper we have focused on the role of PACAP in the mammalian reproductive system.

## Distribution of PACAP in the Hypothalamus

In mammals the distribution of PACAP in the hypothalamus was demonstrated by immunohistochemistry (IHC) (25–30), radioimmunoassay (RIA) (31), sandwich enzyme immunoassay (S-EIA) (32) and *in situ* hybridization (ISH) (30, 33, 34).

Mapping of PACAP required the development of antibodies. The initial antibodies were polyclonal rabbit antibodies. The most potent antibodies were characterized by enzyme immunoassay (EIA) (25) and RIA (31). PACAP in mammals shows the same amino acid sequence (5, 35–37) therefore, antibody against ovine PACAP was able to stain not only in ovine tissues (25, 38), but in many other mammalian species such as rats (26), humans, monkey (27), hamsters, guinea-pigs, ferrets, cats, and pigs (39).

In the hypothalamus IHC revealed PACAP neuronal cell bodies in the supraoptic (SON), paraventricular (PVN), anterior commissural nuclei (ACN), periventricular (Pe) and perifornical regions (Pf), well-defined immunoreactive fiber network in the median eminence (ME) and suprachiasmatic nucleus (SCN) (25, 26, 40). The staining was more prominent in colchicine pretreated rats. PACAP in similar localization was also demonstrated by Kivipelto et al. (28) and Tamada et al. (29). In the ME of intact rats PACAP fibers were mainly found in the internal zone; however, 3 weeks after hypophysectomy the fiber staining appeared in the external zone as well (26), from where PACAP might be released into the pituitary portal circulation. Dow et al. (41) demonstrated that PACAP was present in the hypophysial portal blood of both male and female rats and the amount of PACAP (measured by RIA) was significantly greater than in the peripheral blood. Reversed phase high performance liquid chromatography (HPLC) revealed that the major form in portal blood was PACAP38. The localization of PACAP hypophyseotropic neurons, which send fibers to the portal capillaries, was demonstrated with FuoroGold (FG) tracer injected intraperitoneally (*ip*). From the intraperitoneal space, FG can enter the blood stream and the central nervous system through the ME and the posterior pituitary where the blood-brain barrier (BBB) is missing (42). In control animals FG, spreading in a retrograde manner, appeared in several hypothalamic regions [Pe, arcuate nucleus (ARC), SON and both parvo- (pPVN) and magnocellular portions of PVN (mPVN)]. In pituitary stalk-sectioned rats FG entered the hypothalamus only through the capillary loops of the ME. The stalk section prevented the tracer from entering the hypothalamus through the posterior pituitary. In this model labeling was found in the parvocellular nuclei and the ventral portion of the mPVN. The SON was completely empty. Double labeling revealed that the FG labeled neurons, located in the periventricular area and the ventral portion of mPVN, also showed PACAP immunoreactivity (43). Based on these results the authors concluded that these neurons were hypophyseotropic. Former electron microscopic investigations showed that hypophyseotropic PACAP neurons terminated around the pituitary portal capillaries (29). These neurons may release PACAP into the portal blood.

Hannibal et al. (30, 33) using RIA, IHC and ISH techniques also mapped PACAP in the hypothalamus. RIA revealed that levels of PACAP38 were about 60 times higher than PACAP27 and 10 times higher than the level of PACAP related peptide (PRP). With the use of monoclonal antibodies PACAP and PRP-immunoreactive neuronal perikarya were observed in the medial portion of the pPVN in colchicine pretreated rats. Some PACAP cell bodies were found in the mPVN and the dorsal aspect of the SON. PACAP mRNA containing cells were observed in moderate numbers in the vascular organ of the lamina terminalis, the PVN, the medial mammillary nuclei (MM) and the ventromedial nucleus (VMN).

Moore et al. (34) examined PACAP mRNA expression in the PVN and anterior pituitary of rats using ISH and reverse transcription-polymerase chain reaction (RT-PCR) technique. It was found that PACAP mRNA levels varied significantly during the estrous cycle. PACAP mRNA levels in the PVN declined on the morning of diestrus and started to increase on the morning of proestrus. Highest levels were found 3 h before the proestrous gonadotropin surge then PACAP mRNA level declined again.

**Figure 1:**
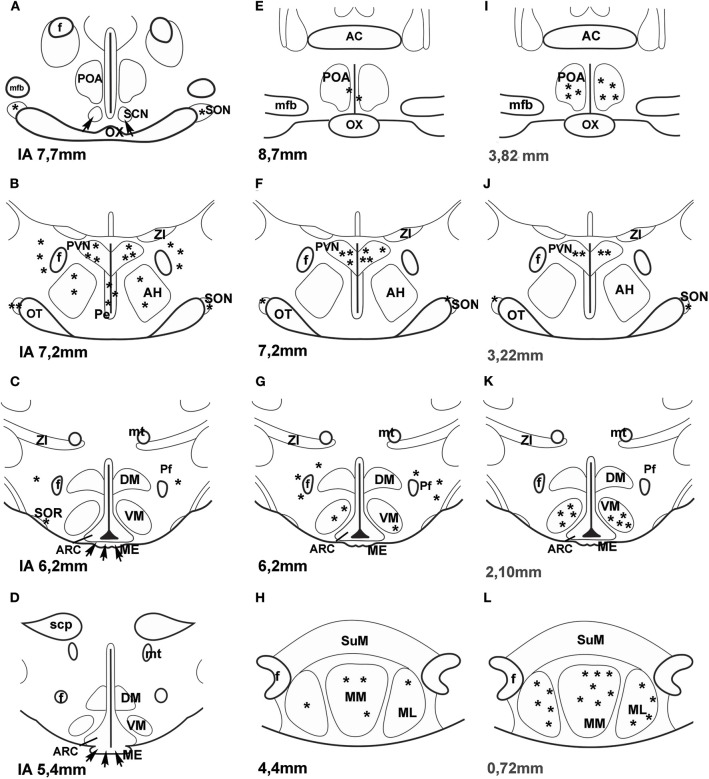
**(A–H)** Distribution of PACAP-immunoreactive cells and fibers and PACAP mRNA- expressing cells in frontal sections of the rat hypothalamus according to Paxinos and Watson's stereotaxic coordinates. **(I–L)** Distribution of PACAP expressing neurons in PACAP-EGFP transgenic mouse hypothalamus according to Franklin and Paxinos' Mouse Brain in stereotaxic Coordinates. **(A–D)** shows PACAP immunoreactive elements in rats. Data were obtained by Köves et al. (26), Kivipelto et al. (28), and Tamada et al. (29). **(E–H)** shows PACAP mRNA expressing cells in rats. Data were obtained by Hannibal et al. (33); Moore and et al. (34) and Dürr et al. (45). **(I–L)** shows EGFP-PACAP in transgenic mice (44). Asterisks indicate cell bodies, arrows indicate fibers. *AC*, anterior commissure; *AH*, anterior hypothalamus; *f*, fornix; *ME*, median eminence; *mfb*, medial forebrain bundle; *ML*, medial mammillary nucleus, lateral part, *MM*, medial mammillary nucleus; *mt*, mammillothalamic tract, *OT*, optic tract; *OX*, optic chiasm; *Pe*, periventricular nucleus; *Pf*, perifornical nucleus; *POA*, preoptic area; *PVN*, paraventricular nucleus; *SCN*, suprachiasmatic nucleus; *scp*, superior cerebellar peduncle; *SON*, supraoptic nucleus; *SOR*, retrochiasmatic portion of SON; *SuM*, supramammillary nucleus; *ZI*, zona incerta. *Reproduced and modified from Köves et al*. *(**46**). Permission to reuse was obtained from Springer Nature. Permission # 4762591108219*.

A few years ago an impressive method was used to map PACAP in the central nervous system of mice. “Transgenic mouse line that harbors, in its genome, a bacterial artificial chromosome containing an enhanced green fluorescent protein (EGFP) expression cassette inserted upstream of the PACAP ATG translation initiation codon” was generated (44). PACAP in the hypothalamus was mainly observed in the PVN, VMN and MM. No green fluorescent protein (GFP) expression was seen in the SON. Figure 1 compares the data, obtained by different methods, concerning the distribution of PACAP protein and PACAP mRNA. It seems that the best correlation is found between mouse EGFP-PACAP (44) and rat PACAP mRNA (30).

There are several lines of evidence that PACAP in the rat hypothalamus colocalizes with some other peptides. Using double labeling immunohistochemistry, Dürr et al. (45) found that PACAP immunoreactivity was present in approximately 20% of pro-opiomelanocortin (POMC) neurons in the ventrolateral part of ARC. These neurons also showed α-melanocyte-stimulating hormone (α-MSH) immunoreactivity. PACAP immunoreactivity was also colocalized with the vesicular acetylcholine transporter (VAChT) in ARC POMC neurons. Vereczki et al. (47) demonstrated that PACAP and VIP immunoreactive cells partially overlapped each other's region in the PVN and SON. Interestingly, in neither cats nor rats do PACAP and VIP immunoreactivities colocalize in the same cells. In spite of the high sequence homology of PACAP and VIP, the two peptides are synthesized in different subpopulations of hypothalamic neurons; however, partial colocalization of PACAP and oxytocin (OT) in the hypothalamic magnocellular neurons of colchicine treated and pituitary stalk sectioned rats was demonstrated. In rats, colchicine treatment and pituitary stalk section enhanced the amount of PACAP and VIP and allowed successful immunostaining in the hypothalamus. PACAP and VIP immunoreactive materials were also stored in different fibers of the posterior pituitary. PACAP fibers formed a dense plexus at the periphery of the posterior lobe, in the vicinity of the intermediate lobe; however, VIP fibers were evenly distributed mainly in the center of the posterior lobe (46, 48).

## PACAP in the Anterior Pituitary

The occurrence of PACAP was observed in both lobes of the pituitary gland. RIA revealed that PACAP levels were much higher in the posterior than in the anterior lobe of the pituitary gland, 270 pmol vs. 3.8 pmol/g wet tissue (31). High levels of PACAP in the posterior pituitary are explained by the fact that PACAP immunoreactive magnocellular neurons send their axons to this part of the gland (25, 26). The number of hypophysiotropic PACAP neurons, those release PACAP to the portal blood, is limited compared to those sending fibers to the posterior pituitary. Most studies using RIA were performed in male rats. There is no available data using RIA to measure levels of PACAP in the anterior pituitary of female rats having various stage of estrous cycle. RT-PCR technique demonstrated that the levels of PACAP in the anterior lobe increased during proestrus (49). In this stage of the ovarian cycle PACAP immunoreactive cells were observed in the anterior pituitary of female rats using IHC. Double labeling showed that PACAP immunoreactivity was present in LH and follicle stimulating hormone (FSH) cells (50). Moore et al. (34) examined PACAP mRNA levels not only in the hypothalamus, but also in the anterior pituitary of female rats. PACAP mRNA levels also varied on the afternoon of proestrus. There was a moderate decrease at the time of the gonadotropin surge (in the afternoon of proestrus between 16 and 20 h) and a significant increase later in the evening. Expression of mRNA encoding follistatin increased significantly following the rise in pituitary PACAP mRNA at the termination of the secondary surge in FSHβ. ISH clearly showed that PACAP expression in the anterior pituitary of male rats was negligible similarly to that of diestrous rats and transiently enhanced in the proestrous stage of female rats. Significantly higher levels were found late evening (20 h) with decrease in the numbers of PACAP expressing cells 2 h later (22 h) (51). IHC was able to reveal PACAP immunoreactivity only in LH and FSH cells, folliculostellate cells (FS) were negative (50). However, RT-PCR analysis of enriched populations of FS did reveal the presence of PACAP in these cells as well (52). The level of PACAP in individual FS cells has to be very low, not enough for immunostaining.

## PACAP Receptors

PACAP shows high sequence homology with VIP therefore binding sites have been characterized on the basis of their relative affinities for PACAP and VIP. Soon after the discovery of PACAP, its receptors were also identified (53–56). The members of the International Union of Basic and Clinical Pharmacology Committee on Receptor Nomenclature and Drug Classification (NC-IUPHAR) subcommittee on receptors for VIP and PACAP agreed on a common nomenclature (57, 58). According to their agreement there are three G-protein coupled receptors: PAC1 (specific for PACAP), VPAC1 and VPAC2 (both of which bind PACAP and VIP with equal affinity). The C-terminal end of PACAP binds to its receptors (59). The receptor is a protein composed of 495 amino acids with seven transmembrane domains (60). Dejda et al. (61) identified regions within the extracellular N-terminal domain of PAC1 which were the major binding sites for PACAP. Three peptide derivates containing a photoreactive p-bensoyl-phenilalanine residue were developed. These photoreactive peptides linked to three fragments of extracellular domains: Ser (62)—Met (63) segment, Ser (64)—Glu (65) dipeptide, and Ser (66)—Met (67).

More detailed analysis using RT-PCR technique with PAC1-specific primers revealed splice variants of PAC1: PAC1s (short form and very short form), PAC1hop1, PAC1hop2, PAC1hip, PAC1hiphop1, PAC1hiphop2, PAC13a and PAC1TM4. Except for the short form (also called null form) the splice variants have inserts in the third intracellular loop. The hip cassette contains 28 amino acids, hop1 28, and hop2 27 amino acids. Stimulation of short and hop1 variants potently increase AC and PLC (3). The very short form lacks 57 amino acids in the first extracellular loop and displays decreased affinity to PACAP27 and PACAP38, but its affinity toward VIP remains the same (68). Blechman and Levkowitz (69) summarized all data concerning the splice variants of PAC1 receptor. An alternative splicing of N-terminal part of the receptor was also found. Alternative splicing alters ligand binding properties and induces different outcomes of the receptor function. A 21-amino acid deletion in the N-terminal extracellular domain resulted in a new splice variant of PAC1 receptor. This domain modulates receptor selectivity and controls the relative potencies of PACAP27 and PACAP38 in PLC stimulation (70).

### PACAP Receptors in the Hypothalamus

In the hypothalamus, PACAP dose dependently stimulates both AC and PLC activities. RT-PCR revealed that in the hypothalamus the major receptor splice variants were PAC1s and PAC1hop2 (71, 72). Joo et al. (73) investigated the distribution of PAC1, VPAC1 and VPAC2 receptors using IHC. In the hypothalamus the most intensive PAC1 labeling was found in ARC, anterior and intermediate Pe, medial preoptic area (POA) and SCN. Occurrence of PAC1 receptors overlaps the major part of the location of gonadotropin hormone-releasing hormone (GnRH) neurons and the termination of the retinohypothalamic pathway in SCN. ISH (74) revealed that PAC1 gene expression showed similarly widespread distribution in the hypothalamus. Autoradiography (75) demonstrated binding sites for PACAP which were not shared with VIP. The most dense and consistent labeling was found in SON and ARC, and moderate presence in SCN, Pe and the lateral hypothalamus by both methods. Additionally, ISH showed labeling in PVN, ventromedial (VMN) and dorsomedial nuclei (DMN). A physiological role of PACAP receptors in the latter two nuclei was also demonstrated (76). Moderate PAC1 receptor mRNA expression was also found in SON and PVN of rat by Nomura et al. (77) using ISH. On the basis of the above-mentioned data it seems that the distribution pattern of PACAP receptors, described by various researchers, depends on the applied methods.

What kind of cells exhibits PAC1 expression or immunoreactivity? The cells exhibiting PAC1 immunoreactivity in the hypothalamus are not fully characterized. Rat brain astrocytes in cell culture exhibit PACAP binding (78, 79), which is associated with proliferation of astrocytes (80). Some research groups (81–83) identified a specific PACAP receptor on astrocytes besides VIP type2 receptor. It was also shown that the VIP neuronal survival effect was partially mediated via PAC hop2 splice variants present in astrocytes (81).

**Figure 2:**
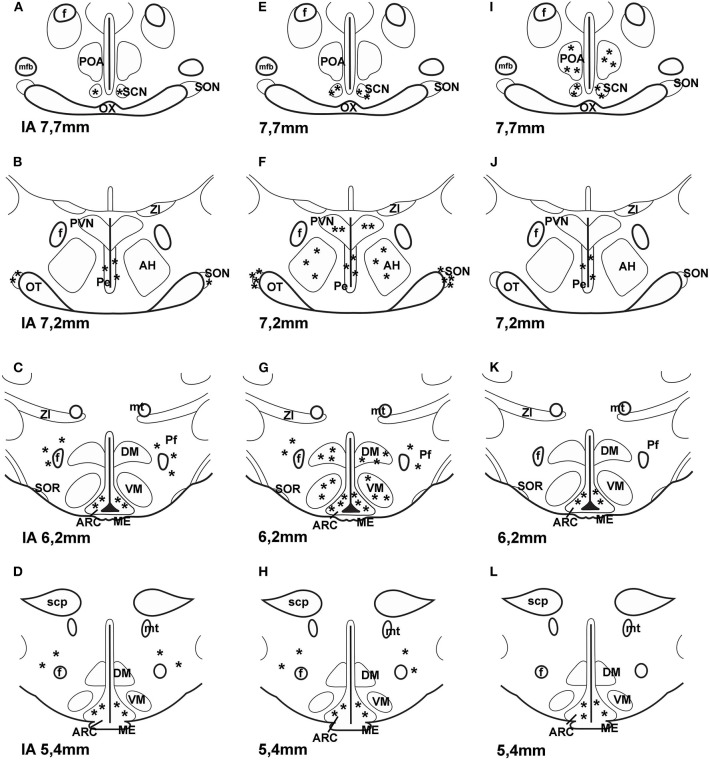
Distribution of PAC1 receptor in frontal sections of the hypothalamus according to Paxinos and Watson's stereotaxic coordinates. **(A–D)** shows data obtained by autoradiography (75). **(E–H)** shows distribution of PAC1 mRNA. Data were obtained by ISH (74, 77). **(I–L)** shows distribution of PAC1 receptor immunoreactivity using IHC (73). Asterisks indicate the place of PACAP binding, or where PAC1 mRNA was expressed or where PAC1 immunoreactivity was detected. *AH*, anterior hypothalamus; *f*, fornix; *ME*, median eminence; *mfb*, medial forebrain bundle; *mt*, mammillothalamic tract; *OT*, optic tract; *OX*, optic chiasm; *Pe*, periventricular nucleus; *Pf*, perifornical nucleus; *POA*, preoptic area; *PVN*, paraventricular nucleus; *SCN*, suprachiasmatic nucleus; *scp*, superior cerebellar peduncle; *SON*, supraoptic nucleus; *SOR*, retrochiasmatic portion of SON; *ZI*, zona incerta. *Reproduced and modified from Köves et al*. *(**46**). Permission to reuse was obtained from Springer Nature. Permission # 4762591108219*.

It is also unclear what the source of PACAP is for PACAP binding sites of astrocytes. Do neurons also express PAC1 receptors? Shioda et al. (84) examined SON and they found PACAP innervation and PAC1 expression on arginine vasopressin (AVP) but not on OT neurons. PACAP was present in noradrenergic fibers from medulla. PAC1 was found on arcuate POMC cells. About 50% of POMC cells express this receptor (45, 85). Later PAC1 and VPAC2 receptor mRNA was also found on neuropeptide Y (NPY) neurons in the ARC (86). It is possible that PACAP receptors are also present on GnRH neurons. Olcese et al. (87) were able to show PACAP receptors on immortalized GnRH neuronal cell lines, but not on processed hypothalamic slides. Nakamachi et al. (88) reported that activity dependent neuroprotective protein (ADNP) in mouse brain colocalized with PAC1 and in the septum and hippocampus ADNP positive cells also exhibited neuron specific enolase (NSE) immunoreactivity. The results obtained by the various methods on the localization of PAC1 receptor in the hypothalamus are summarized in Figure 2.

### PACAP Receptors in the Anterior Pituitary

Soon after the discovery of PACAP, specific binding sites were reported on rat and human pituitary cell membranes (54, 89, 90). ISH and Northern blot analysis revealed a high expression of PAC1 in the anterior and intermediate lobes of the pituitary; however, a very low expression was found in the posterior pituitary (3, 91). Vígh et al. (92) demonstrated that biotinylated PACAP38 could bind to each cell type in anterior pituitary cell culture. About 90% of S-100-positive cells bound biotinylated-PACAP38. A considerable number of GH, PRL and ACTH, and only a few LH, FSH and thyroid stimulating hormone (TSH) producing cells bound PACAP.

All PACAP receptor types including PAC1, VPAC1 and VPAC2 were found in normal rat anterior pituitary tissue and αT3-1 gonadotrope cell lines (93, 94), although PACls and PAClhop1 variants were the dominant forms. Stimulation of short and hop1 variants potently increases AC and PLC as well as intracellular [Ca2+] levels (95).

## Role of PACAP in the Gonadotropic Hormone Secretion of Females

### The Effect of PACAP on the GnRH-LH Axis

#### Hypothalamic Level

At the advent of PACAP research, it seemed that PACAP was a hypophysiotropic factor which stimulated AC in rat anterior pituitary cell cultures (1). *In vivo* experiments were carried out in rats (96–99), mice (100, 101), and sheep (102). As mentioned before, the release of PACAP into rat portal circulation was demonstrated by Dow et al. (41).

Studying the effects of PACAP administration and investigating the lack of PACAP or its specific receptors (PAC1) in certain tissues provide good insight into the role of PACAP in the reproductive functions. The effect of PACAP administration on the gonadotropic hormone secretion varies by dose and route of administration, the studied species, sex, as well as on stages of estrous cycle in females.

##### Intravenous (iv) administration

There are only some experiments where PACAP38 was administered *iv* to female rats before the critical period of proestrous stage of estrous cycle, when the central nervous system prepares itself for the GnRH release. Its effect was examined on plasma LH levels and ovulation. 10 μg *iv* PACAP38 had no effect on plasma LH level and did not interfere with ovulation (96, 97).

##### Intracerebroventricular (icv) administration

In the above-mentioned model 10 μg of PACAP38 administered *icv* before the critical period of the proestrous stage prevented the proestrous LH surge and the expected ovulation on the next morning (96, 103); however, PACAP27 in a same model enhanced plasma LH level and did not influence ovulation (97). The inhibitory effect of PACAP was not a direct action on GnRH neurons; rather it was mainly mediated through corticotropin-releasing hormone (CRH) and endogenous opioids (97, 98). It is possible that CRH directly acts on GnRH neurons because MacLusky et al. (104) have shown interaction between CRH and GnRH neurons in rats. It was also shown that the inhibitory effect of CRH on LH secretion is mediated through endogenous opioids (105). Sawangjaroen and Curlewis (102) used ovariectomized ewes for their experiments. PACAP38 was effective when it was administered *icv* and it depressed the frequency and amplitude of LH pulses. The same research group demonstrated that the inhibitory effect of PACAP was mediated through the medial basal hypothalamus of sheep (106). We have to consider that the migration of GnRH neurons caudally differs among mammals. In rats, GnRH neurons, forming a loose network, are located in the septo-preoptico-anterior hypothalamic area, and the neuronal cell bodies do not reach the medial basal hypothalamus. Only their axons project to the ME (107, 108); however, in humans, similarly to most mammals (including sheep, monkeys, bat, cat, horse, rabbit, guinea pig), GnRH neurons are present in the tuberoinfundibular region as well [reviewed by (109)].

##### Intranasal (in) administration

The effect of many drugs has been studied by the intranasal (*in*) application as well. When PACAP is given *iv* it can cross the blood-brain-barrier (BBB) to a modest degree by way of a saturable transport system (110). It is possible that 10 μg PACAP given *iv* was not enough to prevent ovulation (96, 97). Nonaka et al. (111) demonstrated that PACAP given *in* was effective in improving memory in a very low dose (0.01 μg). In our laboratory, 10 μg of PACAP38 (similar dose which was effective *icv*) was sprayed through the left nostril onto the olfactory region of rats before the critical period of proestrous stage. PACAP prevented ovulation in half of the animals. When ovulation was blocked plasma LH remained at basal levels (99).

##### The effect of PACAP on the onset of puberty

When PACAP was administered to neonatal female rats on the 2nd day of life in a subcutaneous injection (1 μg/animal), it delayed puberty and decreased the number of expelled ova at the first ovulation (112). The intensity of GnRH immunostaining in the septo-preoptico-infundibular system measured in 30-day-old rats was decreased, although there was no difference in the weight of the anterior pituitaries. The pituitary LH content also showed a decrease in PACAP treated rats. PACAP antiserum had a reverse effect on GnRH immunoreactivity. Image analysis supported the light microscopic observations of GnRH immunostaining (112). When PACAP was injected on the 7th day of life, it had no effect on the onset of puberty (46). It seems that neonatal PACAP administration delayed the onset of puberty through the influence of the GnRH neuronal system. It is well-known that the GnRH neurons in mammals derive from the olfactory region (113, 114). Likely PACAP administration on the 2nd day of life does not inhibit, but slows down the migration of the GnRH neurons. But how it is realized, has not been clarified.

Choi et al. (115) examined the role of PACAP and PAC1 in the onset of puberty in female rats. They found that PACAP and PAC1hop1 mRNA in the hypothalamus decreased during the first proestrous day. Disruption of PAC1 synthesis by *icv* administration of a PAC1 antisence oligodeoxynucleotide (ODN) in the late juvenile period considerably decreased GnRH levels in the hypothalamus, GnRH receptor mRNA and LHβ in the anterior pituitary. These alterations induced delay of vaginal opening and first ovulation. It is probable that, in the lack of PAC1 synthesis, PACAP is not effective. In adult rats PACAP mRNA levels rise just before the critical period of the proestrous stage then it decreases during the critical period (34). Without PAC1 receptor in this period, elevated PACAP cannot exert its effect. Because the development of GnRH system is completed by the late juvenile period, it is logical that the onset of puberty is only delayed, not missed.

##### The effect of hypophysectomy on hypothalamic PACAP

Köves et al. (26) demonstrated, that 3 weeks after hypophysectomy, PACAP immunoreactive fibers appeared in the external zone of the ME, although they were not seen in intact or colchicine treated rats. It is possible that under normal physiological conditions PACAP is continuously released into the portal circulation, but this baseline level of the peptide in the ME is insufficient for immunostaining. However, significant decreases in PACAP mRNA and radioimmunoassayable PACAP contents were observed in the hypothalamus 1–2 weeks after hypophysectomy. These decreased levels were restored by administration of GH, PRL, tetraiodothyronine (T4), corticosterone, and testosterone (62).

#### Pituitary Level

Dow et al. (41) clearly showed that hypothalamic born PACAP was released into the pituitary portal circulation. Later it became evident that PACAP mRNA was present in the anterior pituitary (51, 116) and PACAP is synthetized in the gonadotropes (50). The amount of pituitary born PACAP is very low when measured by S-EIA (117), much lower than that of LH (118). Cell immunoblot assay (CIBA) demonstrated release of PACAP from anterior pituitary cells into the culture medium (117). The number of PACAP releasing cells was very low in male and diestrous female rats, and tremendously enhanced in the cultures taken from the pituitaries of proestrous rats late in the evening (20 h) (119). These data clearly show that PACAP is transiently expressed in the anterior pituitary and the number of PACAP releasing cells “in adult rat anterior pituitary cell culture depends on the gender, stage of the estrous cycle in female animals, and on the time of day when the animals were sacrificed” (46). The low amount of PACAP in the anterior pituitary confirms the previous hypothesis that pituitary born PACAP is an auto- and paracrine regulator as it was demonstrated by Radleff-Schlimme et al. (62). Usually two gonadotrope cell lines (Lβ-T2 and αT3-1) derived from transgenic female mice are used to study the effect of drugs on gonadotropin hormone secretion. αT3-1 cells express the α-subunit gene even though they do not express the β-subunit and cannot synthesize LH or FSH, but also express GnRH receptor. Radleff-Schlimme et al. (116) showed that in αT3-1 cell cultures PACAP was released into the medium. This observation well correlates with results showing that PACAP is present in gonadotropes (50) and these cells release PACAP into the cell culture medium (117).

##### The effect of PACAP on gonadotropic hormone release

Soon after the discovery of PACAP Culler and Paschall (120), Hart et al. (121) demonstrated a weak stimulatory effect of PACAP on LH, FSH and α-subunit release in primary pituitary cell cultures. It was also shown that PACAP and GnRH interacted synergistically to stimulate LH release (120). Szabó et al. (119) demonstrated that PACAP could stimulate LH release in pituitary cell cultures derived from proestrous rats. It was visualized by CIBA. This technique is able to show the LH release from individual cells. It was found that “the responsiveness of LH cells to PACAP depends on the gender, on the time of day when the animals were sacrificed and in females on the stage of estrous cycle”. LH cells kept the information received *in vivo*. Those cells taken from the proestrous animals in the morning (10h) were most sensitive to PACAP stimulus.

Kanasaki et al. (122–124) used Lβ-T2 cell lines to investigate the role of PACAP on LHβ subunit release. It was found that PACAP (similarly to GnRH) administered to the cell culture in high frequency pulses, increased LHβ subunit secretion and in low frequency pulses it increased FSHβ subunit secretion. They hypothesize that, under physiological conditions, PACAP contributes to the dynamic control of gonadotropin hormone secretion. In another experiment, PACAP dose-dependently increased cAMP accumulation and increased the basal levels of the α-subunit through the PAC1 receptors and had a synergistic effect on GnRH in αT3-1 cells (125).

##### The effect of PACAP on pituitary gonadotropin gene expression

Tsujii et al. (126, 127) used anterior pituitary cells from adult intact and orchidectomized rats. In a perifusion system, pulsatile PACAP stimulated α-subunit and LHβ mRNA levels but did not affect FSHβ mRNA. By contrast, continuous PACAP increased α-subunit mRNA levels, but suppressed FSHβ mRNA without affecting LHβ mRNA. With the use of αT3-1 cell line it was found that the effect of PACAP on the α-subunit expression was mediated by PAC1 receptor and in part by the cAMP/PKA pathway (128). In other experiments in rat gonadotropes and folliculostellate cells PACAP stimulated follistatin gene expression. PACAP or continuous GnRH downregulated FSHβ mRNA. This action required follistatin (63, 129).

Another research group compared the effect of PACAP and GnRH on gonadotropin gene expression in static pituitary culture and in Lβ-T2 perifused cells (122). In their experiment, high-frequency PACAP pulse preferentially enhanced LHβ gene, whereas low-frequency PACAP pulses specifically enhanced FSHβ gene. This pattern imitated the effect of GnRH pulses. Follistatin gene expression showed similar changes to that of LHβ gene expression; it was increased following high-frequency pulses of either GnRH or PACAP. Low-frequency PACAP pulses enhanced PAC1 expression, whereas high-frequency pulses enhanced GnRH receptor expression.

The above-mentioned results clearly show that the rate of gonadotropin hormone secretion is under a very complex regulation, and the results depend on the circumstances [reviewed by (130)]. In *in vivo* experiments, we have to take into account many other known and unknown factors which may influence the effect of PACAP. The factors may derive from the hypothalamus, from the pituitary itself or from the periphery. Because the cell cultures used in the studies were not taken from different stages of the estrous cycle, we cannot claim that the findings completely mirror the changes in the female pituitary gland.

Table 1 summarizes the data concerning the effect of PACAP38 on LH levels in living animals. Table 2 shows data on the effect of PACAP38 and GnRH on gonadotropin hormone release and gene expression obtained in various cell cultures. It seems that *in vitro* PACAP is basically stimulatory on gonadotropic hormone secretion. As it was mentioned above, our data, obtained by CIBA and pituitary cell cultures, clearly showed that the stimulatory or inhibitory role of PACAP on LH release depends “on the gender, stage of the estrous cycle in female animals, and on the time of day when the animals were sacrificed” (119).

**Table 1:** Effect of PACAP38 on the LH level *in vivo*.

**Route of administration**	**Dose of PACAP38**	**LH**	
		**Female**	
iv	10 μg/250 g rat	–	(131)^*^
icv	10 μg/250 g rat	↓	
icv	10 μg/250 g rat	↓	(97)^*^
in	10 μg/250 g rat	↓	(99)^*^
icv	45 μg/ewe	↓	(102)^**^
		Male	
ia	10 μg/250 g rat	↑	(157)
	30 μg/250 g rat	↑↑	
	100 μg/250 g rat	↑↑↑	
icv	0.8 μg/250 g rat	–	
	8 μg/250 g rat	↑	
	32 μg/250 g rat	↑↑	
iv	10 × 10μg rat	↑	(116)^***^
icv	0.4 μg/100 g rat	↑	(132)

* *Given before the critical period of proestrous stage*.

** *Given to ovariectomized ewes*.

*** *Given in hourly injection*.

*Ia, intraarterial; icv, intracerebroventricular; in, intranasal; iv intravenous*.

*↓ decrease, ↑ moderate increase, ↑↑ high increase, ↑↑↑ very high increase*.

**Table 2:** Effect of PACAP38 and GnRH on the gonadotropic hormone level and gene expression in cell cultures.

**Type of Cell Cultures**	**Examined Parameters**				
Perifused cell culture	α-subunit	LH	FSH		
cPACAP	↑	–	↑		(126, 127)
pPACAP	↑	↑	–		(63, 129)
Primary cell culture	α-subunit	α-subunit			(128)
	mRNA	protein			
PACAP	↑				
GnRH	↑↑	↑↑			
αT3-1 cell line					(128)
PACAP	↑	↑			
GnRH	↑	↑			
Primary cell culture	α-subunit	LHβ	FSHβ	PAC1	(122)
	promoter activity				
cPACAP	↑				
cGnRH	↑	↑	↑		
	mRNA				
cPACAP	↑	↑	↑		
CGnRH + PACAP	↑	↑	↑		
perifused Lβ-T2					
pPACAP high f		↑↑	↑	↑	
pPACAP low f		↑	↑↑	↑↑	
GnRH				↑	

*P, pulsatile; c, continuous; f, frequency*.

*↑ moderate increase, ↑↑ high increase*.

#### Knock Out of PACAP or Its Receptor on Gonadotropin Hormone Secretion

Several research groups generated PAC1 or PACAP knock-out mice (100, 133–135). The mortality of PAC1 null mice was extremely high, 60% in a month after birth. The animals showed serious metabolic disorders. The surviving females showed reduced fertility, but not the males. Normal LH, FSH and PRL staining was observed in their pituitaries (133). Another research group generated both PACAP and PAC1 null mice. Mortality of both PACAP and PAC1 null mice was very high in the first month of life. However, females, that survived beyond this period, exhibited onset of puberty in time. They showed normal estrous cycle. The seminal plug was also normal after pairing. The most strinking abnormality was that only 13% of fertilized eggs were implanted on day 6.5 after mating. Because PRL and progesterone levels were reduced in these animals the authors suggested that the impaired implantation was due to low PRL and progesterone levels (101). Similarly, low birth rates were found by Shintani et al. (135), but they found reduced mating and maternal behavior as well.

### The Effect of PACAP on PRL Secretion

#### Hypothalamic Level

PRL is one of the anterior pituitary hormones. The most prominent role of PRL is to stimulate milk secretion. Later it became evident that PRL was synthetized in many structures other than pituitary. PRL was found in the central nervous system, the immune system, the uterus and in the mammary gland itself and it is accepted that PRL has a multifunctional role [reviewed by (136)].

The effect of PACAP on PRL release is well-established. Miyata et al. (1) found that in superfused cell cultures PACAP stimulated PRL release. Nagy et al. (137) used a special model to examine the effect of PACAP on PRL release. Rat pups were separated from their mother, suckling was suspended. During this refractory period, *iv* injection of PACAP was able to stimulate PRL release in the mothers. However, in sheep PACAP administered *icv* stimulated dopamine release from the tuberoinfundibular dopaminergic neurons and this effect was associated with a suppression of PRL level in the peripheral blood (138). Tohei et al. (139) observed a similar effect after *icv* administration of PACAP to lactating rats exposed to suckling stimuli. PACAP38 decreased PRL secretion and increased the activity of thyrosin hydroxydase (TH) in ME of the pituitary stalk. On the other hand, *icv* injection of PACAP38 did not affect PRL secretion and TH activity in lactating rats, who had their pups taken away, removing the suckling stimuli. Contrary to this, Nagy et al. (137) found that *iv* administration was stimulatory in these same circumstances. It means that PACAP directly stimulated PRL release from the lactotropes.

#### Pituitary Level

PACAP stimulated PRL release from superfused pituitary cells at a very low dose (10^−10^ M) (1). It has been also shown that lactotropes bind biotinylated PACAP38 with high affinity (92). Later, in PRL producing cells three PAC1 variants were detected using RT-PCR method (140). The question arises: what is the source of PACAP for these receptors? Does it originate from the hypothalamus or from the pituitary itself? It seems that PACAP may originate from both sources. Jarry et al. (141) found contrasting *in vivo* and *in vitro* effects of PACAP on PRL release. In the *in vitro* experiments, they used reverse hemolytic plaque assay (RHPA). In this model PRL was inhibited by PACAP. In *in vivo* experiments, they have used medial basal hypothalamus lesioned rats. The lesion destroyed tuberoinfundibular dopaminergic neurons and plasma PRL levels rose. *Iv* administered PACAP further stimulated PRL release. Another research group used a monolayer pituitary cell culture. In this model PACAP inhibited PRL release; however, in aggregated cell cultures or in pituitary fragments, PACAP was stimulatory. This means that a paracrine cell to cell communication is mandatory for the action of PACAP on PRL release. This communicating factor may be interleukin-6 (IL6) which was also stimulated by PACAP in both models (142). PACAP also induced PRL release using AtT-20 and GH3 cell lines (143). Oride et al. (23) summarized the data concerning the role of PACAP in the hypothalamic-pituitary system. They concluded that PACAP alone has a relatively weak simulatory effect on PRL gene expression in lactotropes and PACAP and TRH have a synergistic effect in this regard.

#### PRL and PACAP in Milk

Both PRL and PACAP are present in the milk. The level of PRL in milk is similar to the level found in the general circulation (144). Recently it was shown that dopamine agonists reduce not only milk yield through PRL inhibition, but also mammary epithelial cell activity, survival, and proliferation (145). PACAP was first demonstrated in human milk by Börzsei et al. (123). No significant differences were found in plasma PACAP level of women of different ages or hormone cycles. However, PACAP levels significantly increased in the second and third trimesters of pregnancy and during lactation (64). In milk whey the content of PACAP was 5–20-fold higher than in plasma, and the highest concentration was in colostrum. PACAP content is stable until the tenth month of lactation then it rises again (65). PACAP was also found in the plasma and milk of other mammals such as cow, goat and sheep (146). The source of PACAP, present in the milk, may originate from plasma or from autonomic and sensory nerve endings. PACAP has been shown to be present in nerve fibers, which innervate the smooth muscles of vessels, and in lactiferous ducts and fibers surrounding the alveoli. It was suggested that PACAP immunoreactive sensory fibers might transmit the suckling stimulus to the central nervous system (147). PACAP may also have an effect on mammary epithelium because PAC1 was demonstrated in this tissue using IHC (65). It is not known what the exact function of PACAP in milk is. Tamás et al. (148) hypothesized that “1. PACAP may be essential for the growth and development of newborn; 2. PACAP may be required for the development of the immune system and immunological microenvironment of the gastrointestinal tract; 3. PACAP could be important in the growth and function of the mammary gland.”

#### PACAP in Plasma

PACAP is present in plasma. It was also demonstrated that plasma PACAP38 level increased during the second and the third trimester (64). Lactation moderately enhanced PACAP concentration (149). Kanasaki et al. (123) investigated plasma PACAP levels in human subjects. They found lower PACAP concentration in the second trimester of pregnancy and in several pathological conditions such as premature ovarian failure and idiopathic hypogonadotropic hypogonadism than in normal menstruating women. There is no clear evidence for the source of PACAP in plasma. We exclude the possibility that PACAP may originate from the anterior pituitary. Low PACAP concentration here is only enough for auto- and paracrine actions. Likely magnocellular PACAP neurons sending fibers to the posterior pituitary release a sufficient amount of PACAP into the general circulation (25, 26, 47). PACAP could also originate from sensory nerve endings. Helyes et al. (150) found 2-fold higher PACAP levels in the blood after systematic stimulation of capsaicin-sensitive sensory nerves than in control animals. The most likely option is that PACAP originates from the placenta because PACAP immunoreactive fibers innervate vessels in the uteroplacental unit in humans (151). PACAP38 and PAC1 mRNA has been demonstrated in the placenta as well as immunoreactive stromal and decidual cells in humans and rats (152–155).

## The Role of PACAP in the Gonadotropin Hormone Secretion of Males

At the beginning of PACAP research several research groups investigated the effect of PACAP in adult male rats *in vivo*. They used different routes of administration (*iv, icv* and intraarterial [*ia*]). *Iv* administration of PACAP38 (10 μg/100 g bw) decreased plasma LH levels (156), while its antagonist, PACAP6-38 elevated it. When PACAP38 was repeatedly administered as an *iv* bolus injection (10 × 10 μg), LH concentration was enhanced. Parallel with LH elevation, PACAP mRNA increased seven times in the anterior pituitary. It means that PACAP regulated its own expression (116). The authors supposed that the source of PACAP was the gonadotropes where PACAP exerted an autocrine effect. In another *in vivo* experiment *icv* administration of PACAP (0.4 μg/100 g bw) enhanced LH levels while PACAP6-38 in a same dose decreased it (132). Osuga et al. (157) observed elevations of plasma LH after both *ia* and *icv* administration of PACAP38 (see above Table 1).

Ample experiments were carried out in *in vitro* models (126–128). In monolayer anterior pituitary cell cultures from 7-week-old orchidectomized rats, PACAP attenuated GnRH stimulated LH secretion. When the cells were stimulated by GnRH in a pulsatile manner, continuous presence of PACAP in the culture further enhanced LH, FSH and the α-subunit secretory episodes. This suggests that there is a synergistic effect between the two peptides (126). Later the same research group examined the effect of pulsatile administration of PACAP and found that “pulsatile PACAP stimulated α-subunit and LHβ mRNA level but did not affect FSHβ mRNA. In contrast, continuous PACAP increased α-subunit mRNA level, but suppressed FSHβ mRNA without affecting LHβ mRNA” (127). With the use of a perifused pituitary system PACAP (10 nM) was applied continuously. This treatment induced a rapid and transient release of gonadotropins from pituitary cells of both intact and orchidectomized 7-week-old rats. However, hourly pulsatile PACAP administration in a same dose induced episodic release of LH, FSH and α-subunit, but frequency of these epsiodes gradually decreased. PACAP was a slightly more effective stimulator of LH release by pituitary cells deriving from castrated than from intact rats (127). It was also shown that PACAP stimulated follistatin gene expression in both gonadotropes and folliculostellate cells, and follistatin was required for PACAP to downregulate FSH-β mRNA (129). Kanasaki et al. (122) found a striking difference between the effect of continuous and pulsatile administration of GnRH and PACAP on gonadotropic hormone secretion using Lβ-T2 cell line. The frequency of administration also influenced the effect of the two peptides. High-frequency PACAP pulses enhanced better LHβ gene expression than low frequency pulses and low-frequency pulses enhanced better FSHβ gene expression than high-frequency pulses. The pattern of the effect of PACAP was similar to that of GnRH.

Winters et al. (63) demonstrated that PACAP was able to stimulate α-subunit expression and LH secretion and repress FSH synthesis in fetal male rat pituitary glands as well. Moore et al. (158, 159) revealed a reciprocal relationship between PVN PACAP and FSHβ gene expression in maturing rats. They observed that PACAP and follistatin levels decreased at birth and, as a consequence of it, FSH and the GnRH receptor levels increased. The onset of puberty is also characterized by the increase of FSH synthesis. Later the above-mentioned research group (160) created a transgenic mouse model in which pituitary PACAP was overexpressed. The overexpression was proven by IHC, Western blot, and ELISA analyses. Follistatin, GnRH receptor, and gonadotropin subunit mRNAs were also measured in the pituitary of male transgenic and wild-type mice of various ages using real-time PCR. FSH, LH, and testosterone levels appeared suppressed. In PACAP transgenic mice, in which gonadotropin subunit and GnRH receptor mRNA levels were reduced and the pituitary follistatin expression was increased, the onset of puberty was delayed. After orchidectomy, the testicular negative feedback of pituitary gonadotropin expression remained intact when it was examined in young adult animals (at age 70 days) (160).

## Interactions Between PACAP, GnRH, and Sex Steroids

Lariviere et al. (161) demonstrated that in Lβ-T2 gonadotrope cell line PACAP38 treatment effectively increased intracellular cAMP while GnRH treatment was only mildly effective. In contrast, GnRH very potently enhanced inositol phospholipid turnover and PACAP had a very weak effect. They further investigated the mechanism of the cross-talk between the two peptides (66, 162). It was previously shown that PACAP38 stimulated cAMP via PAC1 receptors (163). Lariviere et al. (162) revealed the molecular mechanism of the cross-talk between PACAP and GnRH in the gonadotropes. They observed that GnRH inhibited the functional coupling of PACAP to the cAMP pathway via novel protein kinase-C (PKC) isoforms. They also demonstrated that GnRH-activated PKC phosphorylated PAC1-R. Grafer et al. (164) found that GnRH stimulated PACAP gene expression in pituitary gonadotropes via multiple signaling pathways acting on CRE/AP-1 sites in the proximal gene promoter.

Ample evidence indicates that there is an interaction not only between PACAP and GnRH, but between these neuropeptides and peripheral sex steroids. It is well-known that ovarian steroids modulate LH secretion. Ortmann et al. (165) pretreated adult female pituitary cell cultures with estradiol, progesterone or both, then added GnRH or PACAP and analyzed the media for LH and cAMP production. Estradiol alone was able to enhance basal LH and cAMP levels, progesterone enhanced only LH, not cAMP. GnRH and PACAP stimuli further enhanced the steroid induced LH release. In another experiment in male rat anterior pituitary cell cultures GnRH treatment enhanced PACAP mRNA expression, dihydrotestosterone (DHT) or progesterone further enhanced this increase; however, DHT or progesterone alone had no effect on PACAP mRNA. On the contrary, estradiol alone depressed PACAP gene expression but did not alter the effect of GnRH on it. Expression of PACAP receptor mRNA was decreased by GnRH treatment, and minimally increased by DHT treatment, but was not altered by the addition of estradiol or progesterone. DHT and GnRH together blunted PACAP receptor gene expression (166). Grafer and Halvorson (167) confirmed the regulatory role of androgens in the function of PACAP. They showed that androgens stimulated rat PACAP promoter-reporter activity in the Lβ-T2 mouse gonadotrope cell line.

A reciprocal interaction between PACAP and ovarian steroids in female rats was also demonstrated in the ovarietomized and sex steroid replacement model. PACAP and PAC1 mRNA expression was enhanced in the medial basal hypothalamus (MBH) and PAC1 mRNA in POA as well upon treatment with progesterone or progesterone + estradiol (168). Németh et al. (169) published data about the effect of sex steroid deficiency on PACAP levels in the central nervous system. Gonadectomy temporarily decreased PACAP38 levels in many regions including the hypothalamus and the pituitary gland of both male and female rats. By 2–3 weeks post gonadectomy PACAP 38 levels were restored and actually continued to increase in the pituitary, surpassing concentrations of those seen in controls by the fourth month after gonadectomy.

The above-mentioned data suggest that there is a regulatory feed-back mechanism between the gonads and the hypothalamo–hypophyseal system. Interestingly, GnRH neurons do not express progesterone, androgen and α-estrogen receptors. Ha et al. (168) suggested that the effect of progesterone on GnRH neurons may be mediated by PACAP neurons. The authors injected PAC1 ODN into the 3rd ventricle. This treatment depressed the progesterone induced enhancement of GnRH mRNA expression. We have to consider that there is no evidence that PACAP neurons exhibit progesterone receptors.

## Interaction Between PACAP and Other Hypothalamic Peptides

Based on documented data in the literature, LH secretion seems to depend on the balance of the amount of GnRH and PACAP. Before the critical period of the proestrous stage, PACAP mRNA in the hypothalamic PVN is enhanced, followed by a decline during the critical period (34). Parallel to this, a large amount of GnRH is released into the portal circulation overwhelming PACAP and inducing LH surge. As GnRH release into the pituitary declines, local pituitary PACAP levels start to rise, now overwhelming GnRH and resulting in cessation of LH release from the anterior pituitary late in the afternoon (46).

PACAP38 regulates GnRH gene expression. Li et al. (156) investigated the effect of *icv* and *iv* administered PACAP38 and its antagonist, PACAP6-38 on GnRH and somatostatin gene expression in the hypothalamus of male rats. *Icv* administration of PACAP induced increases of both GnRH and somatostatin gene expression. PACAP6-38 and *iv* administration of PACAP had an opposite effect. In female rats PACAP applied *icv* or *in* inhibited the proestrous GnRH release. This inhibitory effect may be mediated by the PVN (46), where a majority of CRH neurons are located (170). PACAP increases the hybridization signal of CRH in the PVN (132). The morphological basis of this observation is that CRH fibers establish synaptic contact with GnRH neurons in rats (104) and are found in close juxtaposition with GnRH neurons in the human infundibulum and ME (171). In rats PACAP fibers innervate the paraventricular CRH neurons (172). These observations strongly suggest that PACAP receptors are present on CRH neurons. In concordance with this morphological observation is the finding that CRH antagonist applied *icv* partially prevented the blocking effect of PACAP38 on ovulation (98).

There may be interactions between PACAP and endogenous opioids as well. This hypothesis well correlates with the observation that POMC neurons exhibit PAC1 and VPAC2 receptors in the ARC (45). The inhibitory effect of PACAP on ovulation can be prevented by *ip* administration of naloxone, a general opioid antagonist (98).

About two decades ago it was recognized that kisspeptin (KP) played an important role in the regulation of gonadotropic hormone secretion. In sheep, dynorphin (Dy), neurokinin B (NKB) and KP were co-expressed in some ARC neurons. These cells were named kisspeptin/neurokinin B/dynorphin (KNDy) neurons (173).

KNDy neurons were also observed in mammals other than sheep (174, 175). Ramaswamy et al. (176) demonstrated close interaction between the KP neurons located in the ARC and GnRH neurons in male rhesus monkey using confocal microscopy. KNDy neurons modulate GnRH pulsatile release into the pituitary portal vessels through Kiss1R and subsequent LH release into the circulation. These are the neurons, not GnRH ones, that are the target of ovarian steroids because KNDy neurons exhibit ERα (177, 178). Researchers have come to the consensus that these neurons are what mediate estrogen negative feedback on gonadotropin secretion (179). This cell group is called the GnRH pulse generator. ERα is also expressed by KP neurons located in the POA. These neurons are the place of positive estrogen feedback which is responsible for the GnRH and LH surge (177). ERβ, another estrogen receptor was discovered by Kuiper et al. (180) and a few years later its presence was demonstrated on GnRH cells using ISH, 125I-estrogen binding (181) and IHC (182, 183). Soon after the discovery of KP it became evident that this peptide plays an important role in reproductive functions. Mutation in the KP receptor (*KISS1R /*GPR54*)* leads to hypogonadotropic hypogonadism and infertility in humans (184). KP neurons contain not only ERα but progesterone receptors as well (185).

With the use of Lβ-T2 it was recently demonstrated that KP10 and PACAP stimulated the expression of gonadotropin subunits, and their expression was further increased when KP10 and PACAP were administered together. KP10 increased PAC1 expression. KP neurons may be targets for PACAP in the hypothalamus (186). In a recent study PACAP neurons located in the premammillary nucleus were found to establish direct contact with KP neurons residing in ARC and preoptic Pe. “Targeted deletion of PACAP from the premammillary nucleus through stereotaxic virally mediated cre- injection or genetic cross to LepR-i-cre mice with PACAPfl/fl mice led to delayed puberty onset and impaired reproductive function in female, but not male, mice” (187).

In 2019, Tumurbaatar et al. (188) used immortalized female rat hypothalamic cell lines containing KP expressing neurons from anteroventral Pe (mHypoA-50) or the ARC (mHypo A-55). In both models PACAP enhanced the expression of Kiss1, CRH and neurotensin genes. Its effect was prevented by a protein kinase inhibitor. PACAP was expressed in both cell models and its expression was increased by estradiol.

## PACAP in the Retinohypothalamic Pathway and Its Role in the Circadian Clock

Under normal environmental illumination the LH and FSH plasma hormone levels show cyclic, circadian and circhoral fluctuations. If the illumination is constant, ovulation is interrupted and in rats continuous estrus is found in the vaginal smear. This phenomenon is explained by the “Scharrer hypothesis” (189), which states that photic stimuli from the eye are conducted not only to the main visual centers, but also to some hypothalamic neurons and then to neuroendocrine effector cells. He called this the photoneuroendocrine system. The anatomical basis of this system is the retinohypothalamic tract (190, 191). The retinohypothalamic tract consists of axons of a distinct population of ganglion cells that contain melanopsin and are responsible for non-image-forming photosensation and transmit signals to the brain (192). The main neurotransmitter of the retinohypothalamic tract is the glutamate. The presence of PACAP was demonstrated in this pathway (103, 193) and found to colocalizes with glutamate (194). PACAP, similarly to glutamate, has a light-like effect on circadian rhythms (195). It is generally accepted that interruption of this important pathway induces alterations in the circadian and cyclic rhythms, such as in the ovarian cycle. Studies conducted on rats show that blind animals with heavily degenerated photoreceptors, but with intact non-image forming light perception, have the ability to be synchronized to the light/dark cycle because they have an intact retinohypothalamic tract (196). The main retinorecipient area of the hypothalamus is the SCN which regulates biological rhythms (biological clock). The most characteristic neurotransmitter in this nucleus is VIP. Other important peptides in this area are: histidin isoleucin (PHI), gastrin-releasing peptide (GRP) and AVP. PACAP fibers, present in the retinohypothalamic tract, terminate in the SCN (67). PAC1 receptor mRNA was demonstrated in this region and there was a significant variation in PAC1 mRNA within the SCN and SON during both the light-dark cycle and constant darkness. The expression pattern was similar, but the expression level was higher during constant darkness. Peak levels were observed in the middle of both real and subjective days and nights (197, 198). Mice that are deficient in PAC1 receptors exhibit altered responsiveness of the biological clock to light-induced phase-shifts, but display robust circadian patterns of wheel-running behavior (199). In contrary, mice lacking the VPAC2 receptor, which responds to both PACAP and VIP, indicate that this receptor plays a crucial role in rhythm generation in the SCN. It was also shown that in PAC1 null mice the circadian expression of VIP mRNA in the SCN was altered (200).

It is probable that the effect of PACAP on KP neurons may be mediated by VIP and AVP neurons. Both VIP and AVP were found to activate about half of KP neurons located in the caudal ARC of female mice, but in males just about 10% of these cells (201). KP neurons in the rostral Pe of female mice receive vasopressinergic innervation from SCN, which is thought to play a critical role in the mediation of the circadian signal to GnRH neurons for timing of the proestrous GnRH and consequent LH surge (202).

## Recent Point of View on the Role of PACAP in the Hypothalamo-Hypophysial Gonadotropin System

Experimental data clearly show that PACAP38 is involved in the hypothalamo-hyphyseal gonadotropin regulation. As it was mentioned before, in the last two decades a new regulatory peptide was recognized. KP was discovered in 2001. During the last 18 years it became evident that this peptide plays a crucial role in stimulating GnRH. KP relays steroid hormone negative and positive feedback signals to GnRH neurons. It also stimulates the onset of puberty and relays photoperiodic information to GnRH neurons (203). In Kiss1 knockout male and female mice pulsatile LH secretion is suppressed, gonads are atrophic and puberty does not occur (204). It seems that KP plays an indispensable role as surge and pulse generator. Recent studies have demonstrated that *iv* administration of KP induces ovulation in heifers, where a mature follicle was maintained (205). KP administration, given as a slow constant *iv* infusion of Kp10 (the shortest endogenous form of the KP molecules having biological activity), was also able to stimulate LH secretion and induced ovulation in anoestrus acyclic ewes (206).

**Figure 3:**
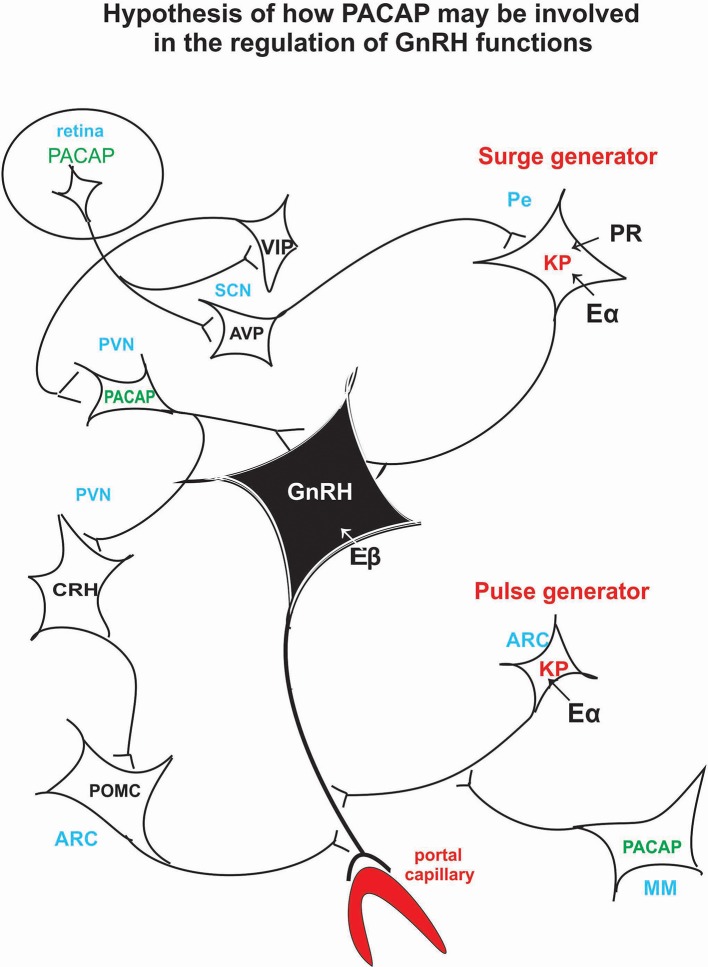
Schematic illustration of the hypothetical pathway of how PACAP may be involved in the regulation of the GnRH release. Retinal PACAP may influence AVP and VIP cells located in the suprachiasmatic nucleus (biological clock). AVP fibers terminate on KP neurons which create the surge generator. PACAP neurons, located in PV, may receive information from the suprachiasmatic VIP neurons. PACAP may exert its effect directly on GnRH cell bodies or via CRH and POMC neurons on GnRH axons. PACAP neurons residing in MM may influence KP neurons which create the “pulse generator.” ARC, arcuate nucleus; AVP, arginine vasopressin; CRH, corticotropic hormone-releasing hormone; Eα, estrogen receptor alpha; Eβ, estrogen receptor beta; GnRH, gonadotropic hormone-releasing hormone; KP, kisspeptin; MM, medial mammillary nucleus; PACAP, pituitary adenylate cyclase activating polypeptide; Pe, periventricular area; POMC, proopiomelanocortin; PR, progesterone receptor; PVN, paraventricular nucleus; SCN, suprachiasmatic nucleus; VIP, vasoactive intestinal polypeptide.

PACAP knockout experiments suggest that PACAP is modulatory rather than mandatory in reproductive functions, and it has a fine tuning role in the regulation of gonadotropin hormone secretion. Its hypothalamic level decreases just before the critical period of proestrous stage (34) then GnRH starts to release into the portal blood. If we artificially enhance hypothalamic PACAP levels by *icv* administration (97), it inhibits the effect of KP and GnRH. Recent experiments show that in Lβ-T2 cells there is an additive effect of KP and PACAP on gonadotropic hormone secretion (186). There is evidence that both KP and PACAP are released into the portal circulation (41, 207); however, it is not clear what the impact of this is. An *in vivo* experiment, when KP alone or KP and PACAP together administered *icv* to proestrus rats before the critical period, would help elucidate whether KP could induce ovulation when blocked by another agent for example by pentobarbital or PACAP. This experiment would give more insight into the role of these peptides. Figure 3 schematically illustrates the hypothetical pathway of how PACAP may influence GnRH release. PACAP, present in the retinal ganglion cells, may relay photic stimuli through AVP and KP neurons to the GnRH neuronal cell bodies residing in the septo-preoptico area. PACAP, present in the PVN, may receive information from suprachiasmatic VIP cells and may act directly on the septo-preoptico GnRH neuronal cell bodies or through CRH and POMC neurons on GnRH terminals. PACAP neurons located in MM region may influence GnRH release into the portal circulation via arcuate KP neurons.

Concerning the role of pituitary born PACAP, we only have data from *in vitro* experiments carried out by Kanasaki et al. (123, 124). Under physiological conditions “the locally produced pituitary PACAP and its receptor (PAC1) may be involved in the GnRH pulse frequency-dependent gonadotropin subunit gene expression”. Pituitary born PACAP influences the responsiveness of gonadotropes to GnRH (119) in an autocrine and paracrine manner (116). The roles of PACAP on the hypothalamo-hypophysial gonadotropin system are as follows:

1. In physiological conditions PACAP in the hypothalamus of males is mainly stimulatory and in females it is inhibitory on gonadotropin hormone secretion.
2. PACAP delays puberty in both genders.
3. Pituitary-born PACAP is an auto- and paracrine factor. It is stimulatory in males. In females it is stimulatory on gonadotropin hormone secretion in the morning of proestrus, but inhibitory in the afternoon of proestrus.
4. It seems that PACAP and PAC1 knockout leads to impared fertilization. This implies that PACAP is necessary for the full implantation of fertilized ova. In this sense PACAP and PAC1 knockout conditions are very similar to the effect of ERβ knockout on reproduction. PACAP knockout reduces mating and maternal behaviors as well.

## Significant Gaps in Research

Reviewing the data concerning the effect of PACAP on pituitary hormone release, it seems that the researchers mostly used pituitary cell cultures from male animals. It is clear that there is a sexual dimorphism in the responsiveness of pituitary cells to PACAP. This is the reason why it is difficult to draw a clear conclusion from the divergent data. The other difficulty is that in some experiments immortalized pituitary cells were used to study the effect of PACAP on hormone release and gene expression. These cell lines are removed from their natural environment and other factors are eliminated which are present in the pituitary *in vivo*.

There are many *in vitro*, but not enough *in vivo* experiments with PACAP38. Fournier et al. (208) summarized the modification of PACAP and the consequence of modifications for the binding properties and biological activity of the analogs. The main need is to produce stable, potent and selective agonists. However, the results also depend on the tissue used and the presence of PAC1 splice variants existing in the selected tissue. The use of PACAP in clinical treatment needs stable analogs because the half-life of the natural PACAP in the blood circulation is only 5–10 min (209) and only a very small amount, about 0.05% of the *iv* injected dose enters the central nervous system from the blood circulation (210, 211).

## Author Contributions

KK, ÁC, ES, OK, AH, and FS have collected data in the literature in order to give timely and accurate review.

### Conflict of Interest

The authors declare that the research was conducted in the absence of any commercial or financial relationships that could be construed as a potential conflict of interest.

## Abbreviations

AC: adenylate cyclase
ACN: anterior commissural nucleus
ACTH: adrenocorticotropic hormone
ADCYAP: adenylate cyclase activating polypeptide
ADNP: activity dependent neuroprotective protein
αMSH: α-melanocyte-stimulating hormone
ARC: arcuate nucleus
AVP: arginine vasopressin
BBB: blood-brain barrier
c-AMP: cyclic adenosine-monophosphate
CIBA: cell immunoblot assay
CRH: corticotropin hormone-releasing hormone
DHT: dihydrotestosterone
Dy: dynorphin
EGFP: enhanced green fluorescence protein
EIA: enzyme immunoassay
ERα: estrogen receptor α
ERβ: estrogen receptor β
FG: FluoroGold
FRF: follicle-stimulating hormone-releasing factor
FS: folliculostellate cell
FSH: follicle-stimulating hormone
GFP: green fluorescence protein
GH: growth hormone
GnRH: gonadotropin hormone-releasing hormone
GRP: gastrin releasing peptide
HPLC: high pressure liquid chromatography
IHC: immunohistochemistry
*ia*: intraarterial
*icv*: intracerebroventricular
*in*: intranasal
*ip*: intraperitoneal
*iv*: intravenous
ISH: *in situ* hybridization
KNDy: kisspeptin/neurokinin B/dynorphin
KP: kisspeptin
LH: luteinizing hormone
MBH: medial basal hypothalamus
ME: median eminence
NKB: neurokinin B
NPY: neuropeptide Y
NSE: neuron specific enolase
ODN: oligodeoxynucleotide
OT: oxytocin
PACAP: pituitary adenylate cyclase activating polypeptide
PCR: polymerase chain reaction
Pe: periventricular region
Pf: perifornical region
RHPA: reverse hemolytic plaque assay
PHI: peptide histidine isoleucine
PKC: protein kinase C
PLC: phospholipase-C
POA: preoptic area
POMC: pro-opiomelanocortin
PRL: prolactin
PRP: PACAP related peptide
PVN: paraventricular nucleus
mPVN: magnocellular portion of PVN
pPVN: parvocellular portion of PVN
RIA: radioimmunoassay
RT-PCR: reverse transcription-polymerase chain reaction
SCN: suprachiasmatic nucleus
S-EIA: sandwich enzyme immunoassay
SON: supraoptic nucleus
T4: tetraiodothyronine
TSH: thyroid stimulating hormone
VAChT: vesicular acethycholine transporter
VIP: vasoactive intestinal polypeptide
VMN: ventromedial nucleus.
